# Fourier transform power spectrum is a potential measure of tissue alignment in standard MRI: A multiple sclerosis study

**DOI:** 10.1371/journal.pone.0175979

**Published:** 2017-04-17

**Authors:** Shrushrita Sharma, Yunyan Zhang

**Affiliations:** 1 Biomedical Engineering Program, Faculty of Graduate Studies, University of Calgary, Alberta, Canada; 2 Hotchkiss Brain Institute, University of Calgary, Alberta, Canada; 3 Department of Clinical Neurosciences, University of Calgary, Alberta, Canada; 4 Department of Radiology, University of Calgary, Alberta, Canada; Nanjing Normal University, CHINA

## Abstract

Loss of tissue coherency in brain white matter is found in many neurological diseases such as multiple sclerosis (MS). While several approaches have been proposed to evaluate white matter coherency including fractional anisotropy and fiber tracking in diffusion-weighted imaging, few are available for standard magnetic resonance imaging (MRI). Here we present an image post-processing method for this purpose based on Fourier transform (FT) power spectrum. T_2_-weighted images were collected from 19 patients (10 relapsing-remitting and 9 secondary progressive MS) and 19 age- and gender-matched controls. Image processing steps included: computation, normalization, and thresholding of FT power spectrum; determination of tissue alignment profile and dominant alignment direction; and calculation of alignment complexity using a new measure named angular entropy. To test the validity of this method, we used a highly organized brain white matter structure, corpus callosum. Six regions of interest were examined from the left, central and right aspects of both genu and splenium. We found that the dominant orientation of each ROI derived from our method was significantly correlated with the predicted directions based on anatomy. There was greater angular entropy in patients than controls, and a trend to be greater in secondary progressive MS patients. These findings suggest that it is possible to detect tissue alignment and anisotropy using traditional MRI, which are routinely acquired in clinical practice. Analysis of FT power spectrum may become a new approach for advancing the evaluation and management of patients with MS and similar disorders. Further confirmation is warranted.

## 1. Introduction

The alignment and integrity of nerve fibers are associated with the conducting efficiency of nerve signals in the brain. In many neurological diseases such as multiple sclerosis (MS), the usual coherency of nerve fibers is disrupted following tissue injury. This disruption is shown to occur not only in focal plaques of MS, but also in the normal appearing white matter (NAWM) [1], leading to paramount functional impairments in patients [2]. A number of studies have attempted to measure white matter coherency using magnetic resonance imaging (MRI); however, there are few methods based on clinical MRI protocols. The availability of a method clinically has important implications both in the evaluation and treatment of nerve pathology.

Current MRI methods for assessing white matter coherency are mainly based on advanced MRI techniques, such as diffusion-weighted imaging [3,4]. Diffusion-based MRI evaluates the random movement activity of water molecules in a tissue. Through assessment of both the magnitude and direction of water diffusion, diffusion tensor imaging (DTI) including fractional anisotropy (FA) and directional diffusivity can detect the organizational property of specific fiber tracks that particularly dominates the white matter [5]. Recent studies have also found that the potential of DTI can be significantly enhanced with combination with image post-processing strategies [6,7]. Indeed, Stamile et al show that histogram analysis of DTI fractional anisotropy is more sensitive to subtle tissue changes in MS lesions than traditional regions-of-interest approaches and can differentiate nerve fibers with or without changes within the same bundle [7]. On the other hand, a new MRI method named susceptibility tensor imaging has also shown promise for detecting white matter alignment [8]. This method uses specific MRI pulse sequences that are sensitive to magnetic susceptibility generated in a tissue, where a 3-dimensional susceptibility tensor is modeled. Using both animal and human brains, Li and colleagues demonstrate that susceptibility anisotropy relates to the orientation of myelin in white matter and is significantly decreased in mouse with dysmyelination. However, while with great potential, most of the aforementioned techniques require advanced image acquisition and assessing skills, are not routinely obtained in clinical practice in many diseases, and their utility are subject to further validation.

An alternative approach for assessing tissue anisotropy is to use image post-processing methods. In this regard, spatial frequency-based methods play an important role such as the Fourier transform (FT). The FT identifies the total content of frequencies enclosed in an image. In the frequency domain, the pixels tend to group along the direction of tissue alignment and form shape-specific clusters; circular cluster represents isotropic tissue and elliptical cluster refers to anisotropic tissue. Based on this theory, Begin et al. [9] have successfully detected the orientation of myelin using histological images from demyelinated spinal cord of mouse. Similarly, using fixed hippocampal specimens from healthy human brain, Nazaran et al. [10] demonstrate that the dominant orientation of an angular histogram based on 2D FT corresponds to the alignment of myelinated fibers in histology. In addition, multiple other studies have indicated the enhanced potential of orientation metrics derived from the power spectrum of FT. Some demonstrates the ability of Fourier power spectrum for identifying the weave pattern and aligning stripes in natural fabrics [11,12]. Others [13,14] show that the density and orientation of FT power spectra are highly correlated with the organization of collagen fibers in mice with fibroblasts. These studies highlight the potential of FT-based image analysis methods, although demonstration of their utility in clinical images is limited.

In this study, based on the power spectrum of 2D FT, we developed a new approach for assessing tissue alignment using T_2_-weighted MRI. This method is validated using a highly aligned brain white matter structure, corpus callosum. To test the feasibility of this method for clinical use, we also compared results from healthy controls with those from patients with mild and advanced MS.

## 2. Materials and methods

### 2.1 Subjects

This is a retrospective study based on MR images acquired from 19 MS patients and 19 age- and gender-matched controls. Of the 19 patients, 10 had relapsing-remitting MS (RRMS) and 9 had secondary progressive MS (SPMS). The mean (standard deviation) age was 37.5 (12.5) years for RRMS and 59.5 (10.5) years for SPMS. The focus of the original clinical study was to assess the structure and function of corpus callosum, a white matter structure coordinating the conduction of numerous inter-hemispheric functions [15]. To understand the difference between cohorts, the recruitment was done in a way such that only patients with very mild RRMS (disability score ≤ 3/10) or very advanced SPMS (disability score ≥6/10) were included. This study was approved by the Institutional Health Research Ethics Board. Written informed consent was obtained from all participants.

### 2.2 MRI acquisition

All images were acquired at a single 3T MR system (GE Healthcare, DISCOVERY MR750, Milwaukee, USA). Whole brain MRI protocols included both T_1_- and T_2_-weighted sequences based on clinical standard. In this study, we focused on T_2_-weighted MRI because that can sensitively detect tissue abnormalities without the need for contrast injection. These images were obtained with a spin-echo sequence with field of view = 240 x 240 mm^2^; matrix size = 256 x 256; repetition time (TR)/echo time (TE) = 6035/83 ms; and slice thickness = 3 mm, without gap. Acquisition of T2-weighted MRI took 2 minutes per subject.

### 2.3 Selection of image and regions of interest

**Fig 1:**
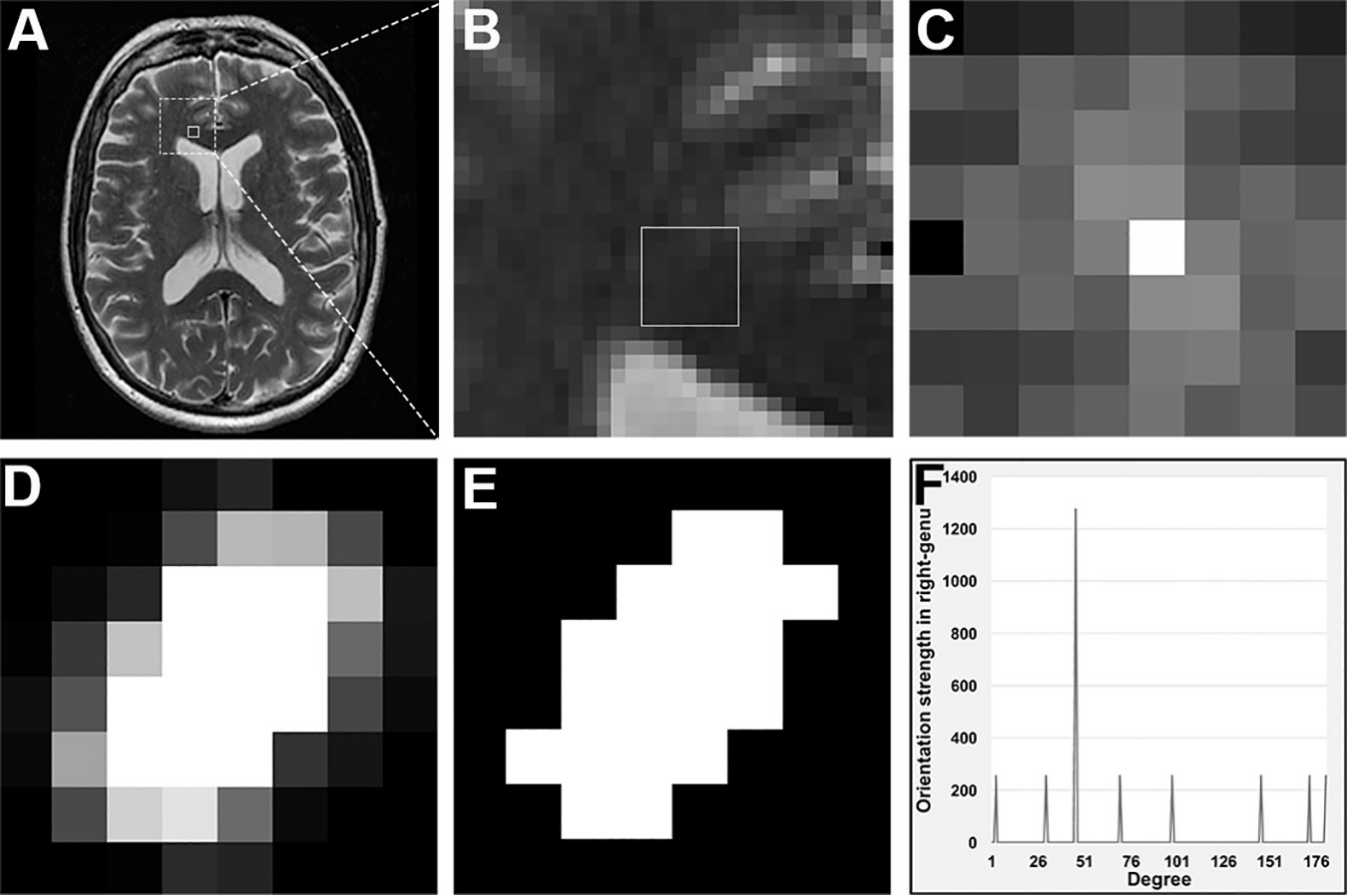
Method demonstration. Shown are an example ROI in the right genu of corpus callosum (A, small box); Zoomed view of the ROI (B); and Fourier transform of the ROI (C). Then, Fourier transform power spectrum of the ROI is shown after normalization (D) and thresholding (E). Based on E, the orientation profile of the ROI is calculated (F), from which corresponding angular entropy can be computed.

**Fig 2:**
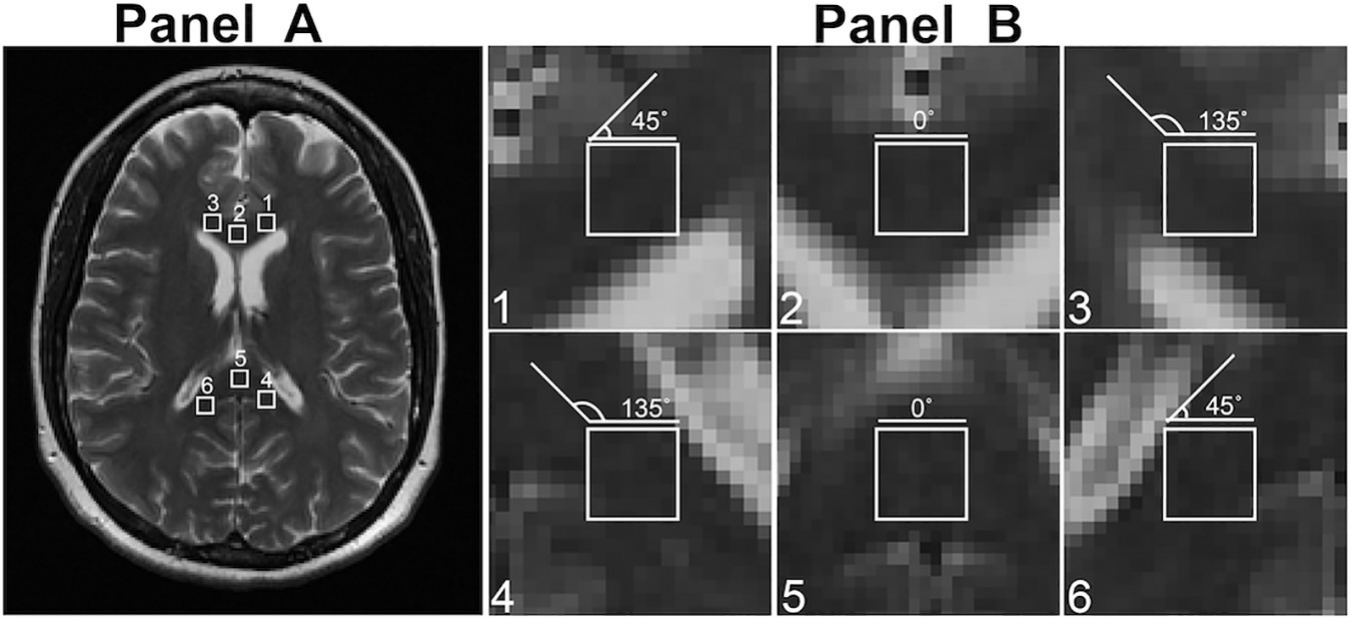
Representative ROIs along with predicted major aligning orientations based on the anatomy of corpus callosum. Panel A shows an example T_2_-weighted MR image from a control subject, where 6 ROIs are highlighted, located respectively in the left (1), central (2), and right (3) aspects of the genu and splenium (4, 5, 6). The predicted major orientations of these ROIs are: ROI 1 = ROI 6 = 45°; ROI 2 = ROI 5 = 0°; and ROI 3 = ROI 4 = 135°, similar to the trajectory direction of specific fiber tracks. Note that the same angle between paired ROIs (1 versus 6; 2 versus 5; 3 versus 4) reflect a near parallel trajectory direction of fiber tracks going through correspondent ROIs.

In both patients and controls, we examined T_2_-weighted MR images that demonstrated the best delineation and the largest area of corpus callosum at equivalent regions, both for the genu and splenium of the structure. To include image areas with different aligning directions, six regions of interest (ROIs) were selected from the axial images of the brain, located at the left, central, and right aspects of genu and splenium respectively (Fig 1). The dominant aligning angle of these ROIs can be visually observed and were estimated at 45°, 0° and 135° at left, central and right genu and 135°, 0°, 45° at left, central and right splenium respectively (Fig 2). The size of these ROIs ranged from 6x6 to 8x8 pixels, equivalent to 31.64 mm^2^ to 56.25 mm^2^; large ROIs were located in the central regions of the genu and splenium where showed the largest cross-sectional area of the corpus callosum. Based on our preliminary tests, ROIs smaller than the proposed size could not provide sufficient content of directional information whereas ROIs larger than that could not fit into the boundary of the structure. To keep consistency, MR images that involved focal lesions within or crossing the corpus callosum were excluded. In addition, patients with the corpus callosum being too atrophic to fit any ROIs were also excluded.

### 2.4 Analysis of tissue alignment

Our orientation analysis method involved several image processing steps. These included: 1) convert identified images into the frequency domain; 2) calculate power spectrum; 3) extract angular distributions of the power spectrum; and 4) compute the dominant orientation and directional complexity in a ROI. We used a fast Fourier Transform (FT) algorithm (ImageJ, NIH, version 1.8, USA) to obtain the frequency content in each ROI.

**Fig 3:**
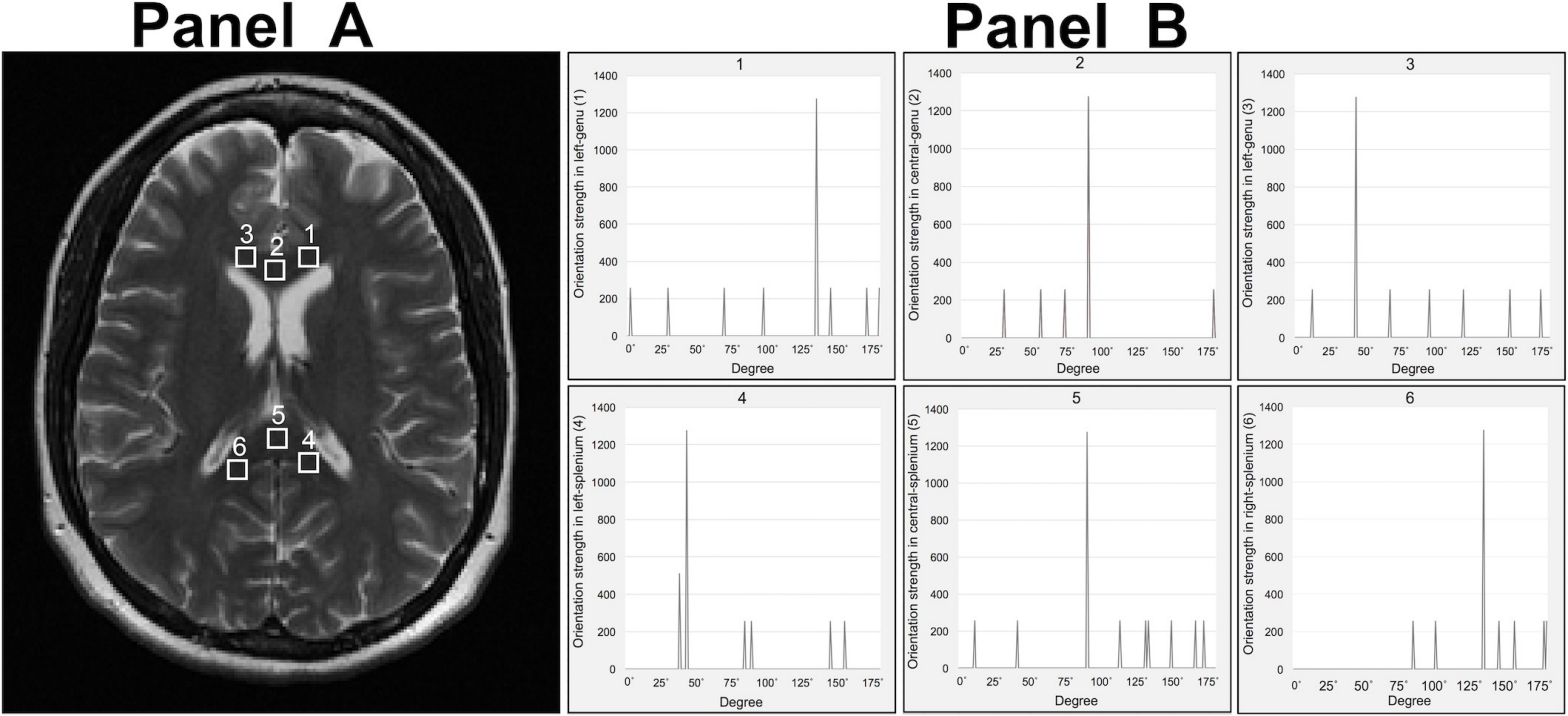
Examples of calculated orientation profiles using our method from chosen corpus callosum ROIs. Panel A shows the same T_2_-weighted image and ROIs as seen in Fig 2. Panel B shows the major (the highest peak) and all aligning directions in each ROI in the frequency domain, which are perpendicular to the angles shown in Fig 2. Here, ROI 1 = ROI 6 = 135°; ROI 2 = ROI 5 = 90°; and ROI 3 = ROI 4 = 45°, 90° from the direction of fiber tracts.

Further analysis of these images was done using algorithms built in-house. To enhance frequency energy, we calculated the power spectrum of the FT, the power spectrum was then normalized through computation of its logarithmic value and thresholded to eliminate noise and edges. Eventually, the upper twenty percentile (80–100) of the intensity of normalized power spectrum was preserved. Next, polar conversion was applied to the thresholded power spectrum, from which the distribution of the orientation angles was obtained for each ROI; the higher the peak, the greater aligning strength at that angle. The angular location of the highest peak in an orientation distribution was considered the dominant direction of the tissue contained in a ROI (see plots in Figs 1 and 3).

### 2.5 Analysis of alignment complexity

Based on the angular distribution profile of a ROI, we also calculated another outcome named angular entropy. It is a measure of angular scattering of tissue orientations and thus reflects the complexity of tissue alignment. Angular entropy (ε) was calculated as ε = -Σ p_θ_*{log p_θ_}, where p_θ_ referred to the probability of alignment at a certain angle θ. Based on this equation, the angular entropy gave rise to a negative value, with zero being the maximum.

### 2.6 Statistical analysis

We used a mixed-effect modeling method to evaluate differences in tissue complexity using a statistical package Stata (StataCorp, Texas, USA; version 12). This method allowed us to consider both intra-subject variances between different ROIs and inter-subject variances between control and patient groups. Tissue angular entropy was defined as the dependent variable. In the analysis of dominant orientations of a tissue, we compared the derived angles using our method with angles estimated based on anatomy within individual ROIs. This correlation was also done using mixed-effect modeling in Stata. For all of the comparisons, P-value < = 0.05 was defined as significance.

## 3. Results

### 3.1 Sample characteristics

We examined 204 corpus callosum ROIs in total, 90 from patients and 114 from controls. Of the 90 patients ROIs, 35 were in SPMS from 9 patients, and 55 in RRMS from 10 patients. Anatomically, there were 14, 15, and 15 patient ROIs located in the left, central, and right aspects of genu, and 15, 16, 15 in the left, central, and right aspect of splenium. Please see Table 1 for details. In controls, 6 ROIs per subject were identified in the corpus callosum, totalling 19 subjects. Overall, 24 ROIs were excluded from patients due to excessive tissue atrophy; most of these regions were located in the left (4) and right (4) genu of SPMS patients. No lesions were involved in any of the remaining ROIs, and all control ROIs appeared normal in MRI.

**Table 1:** The number of regions of interest examined in the corpus callosum per group.

	Control	RRMS	SPMS	Patients	Total
Left Genu	19	9	5	14	33
Central Genu	19	9	6	15	34
Right Genu	19	10	5	15	34
Left Splenium	19	9	6	15	34
Central Splenium	19	9	7	16	35
Right Splenium	19	9	6	15	34

### 3.2 Correspondence of dominant tissue alignment between quantified and observed angles

**Fig 4:**
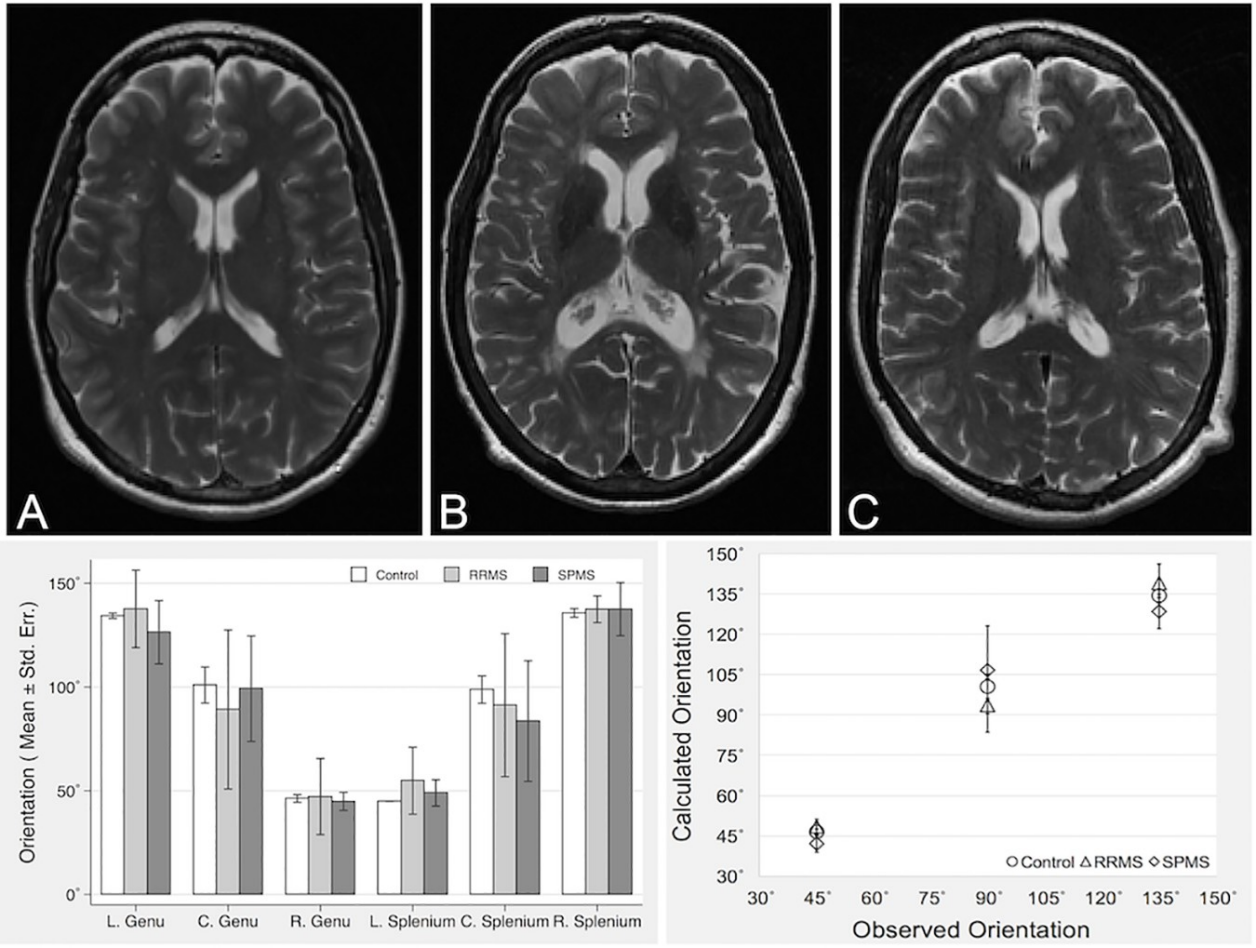
Summarized dominant directions per ROI and subject group based on T_2_-weighted MRI of corpus callosum. Top row shows example images of corpus callosum from a control subject (A), and from patients with relapsing-remitting (B) and secondary progressive (C) MS, suggesting increasing degrees of brain atrophy. Bottom left plot shows the dominant aligning angles of each ROI from the 3 groups. Bottom right plot shows the correlation between predicted and calculated dominant orientations at each aligning angle. Data shown are mean and standard error in plots (L: left, C: centre and R: right).

From analysis of the polar-converted FT power spectrum, we detected a spectrum of orientation peaks per ROI, in which a dominant peak was persistently identified in each ROI (Fig 3). The mean (± standard error) angles of the dominant peaks were 134.32° (0.684°), 100.63° (4.58°) and 46.42° (0.977°) in the left, central and right genu, and 45° (0°), 97.74° (3.41°) and 135.79° (1.1°) in the left, central and right splenium respectively in the frequency domain. Based on the anatomical location of the fiber tracks of the corpus callosum, the dominant orientation of the 6 ROIs were paired with each other in 3 groups: left genu showed similar angles to right splenium (134.32° versus 135.79°); right genu with left splenium (46.42° versus 45°), and both central ROIs of genu and splenium were parallel with each other (100.63° versus 97.74°). Following perpendicular conversion of these angles based on the reciprocal Theorem of the FT, these dominant angles corresponded to 44.32°, 10.63° and 136.42° in the left, central and right genu, and 135°, 7.73° and 45.79° in the left, central and right splenium in image domain. Such converted orientations were significantly correlated with the observed directions based on the anatomical location of the corpus callosum, with an overall correlation coefficient of 0.8976 (p = 0.008), where 0.9564 for controls; 0.8537 for RRMS patients and 0.8075 for SPMS patients (Fig 4).

### 3.3 Increased tissue angular entropy in patients as compared to controls

**Fig 5:**
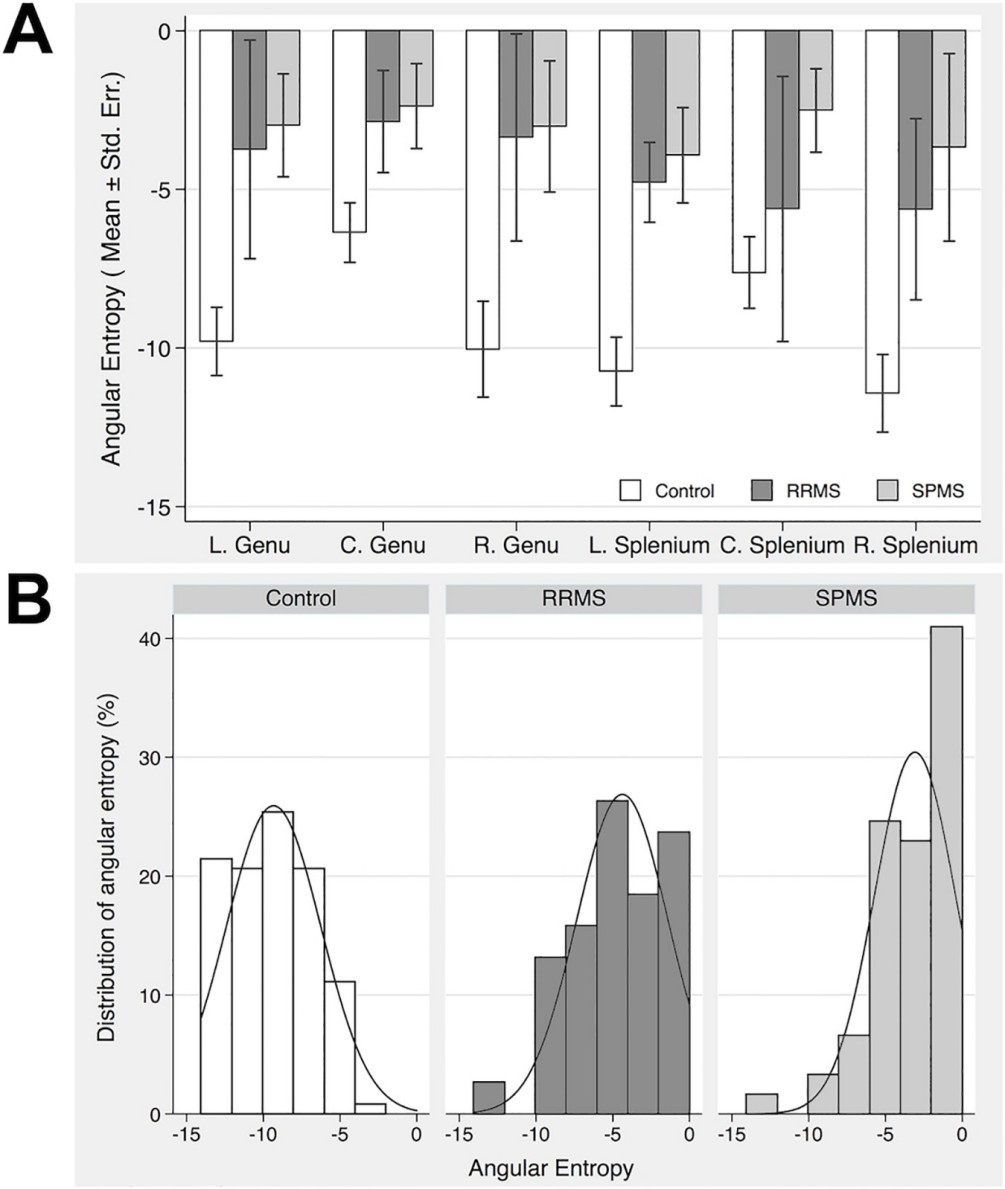
Angular entropy in each ROI of the 3 subject groups. Panel A shows the mean and standard error of angular entropy summarized by group and ROIs, and panel B demonstrates the distribution of angular entropy of all ROIs per group according to the severity of angular entropy. Note that large values close to 0 refer to high angular entropy, thus high tissue complexity. In panel B, the peak location of the distribution curves shifts toward lower values of angular entropy (less negative) from control to RRMS and then to SPMS, representing greater tissue complexity and injury in patients, particularly in those with SPMS (L: left, C: centre and R: right).

Based on mixed-effect modeling, we found that the angular entropy in MS patients was significantly higher (less negative) than in control subjects (mean ± standard error = -3.6785 ± 0.8361 versus -9.4278 ± 0.5986, p = 0.013), and tended to be higher in SPMS than in RRMS patients (mean ± standard error = -3.2106 ± 0.8416 versus -4.1464 ± 0.8305, p = 0.069). This pattern was consistent across all 6 ROI locations (Fig 5A). To understand the heterogeneity of tissue structure within a group, we also evaluated the distribution of angular entropy from all ROIs (Fig 5B). As shown by individual fitting curves, the peak location was -9.4335 in controls, significantly different than -4.1589 and -3.2958 in RRMS and SPMS groups. In fact, the lowest angular entropy was measured at -14.0464 from a ROI in controls, and the highest at 0 from a ROI in SPMS.

## 4. Discussion

In this study, we presented a new image processing method for assessing tissue alignment and anisotropy based on FT power spectrum. Using the highly oriented corpus callosum as a validation model, we show that tissue alignment derived from our method is correlated with the anatomical orientations of this structure. Furthermore, through quantitative analysis of the aligning complexity of corpus callosum, we further demonstrate that the current method may have the potential to detect subtle structure disruptions in a lesion-free area of patients using conventional MRI.

It is well known that the power spectrum of FT provides intensified information of an image. Mathematically, the power spectrum calculates the square of the spectral energy contained in an image [13]. This property facilitates a dramatic increase in both the contrast and detectability of power spectrum. Indeed, using a mouse model of MS (experimental autoimmune encephalomyelitis), a recent study has shown the ability of FT power spectrum to detect the anisotropy of demyelinated axons in mouse spinal cord [9]. However, that study was done using histological images that have ultra-high resolution and the alignment of nerve fibres are already visible using bare eyes. In the current study, we took advantage of the benefit of several image pre-processing procedures including spectral normalization and thresholding besides calculation of the power spectrum. Together with polar conversion of corresponding power spectra, unique alignment characterises of a ROI were generated using standard MRI. This could be useful in future clinical studies when assessment of focal tissue structure is required without the acquisition of additional advanced imaging sequences.

To test the validity of our method, we used corpus callosum of the brain as an evaluation model. Corpus callosum is a unique interhemispheric structure that contains highly organized white matter fibers. According to the literature, different regions of corpus callosum follow different white matter trajectories, facilitating the conduction of specific brain functions [16]. Specifically, the genu connects anterior aspects of the brain between hemispheres allowing for sensory and motor functions, and the splenium links primarily the occipital lobes to coordinate visual function [17]. In anatomical MRI, these fiber bundles appear projecting at different angles as predicted in Fig 2. When focusing on a local ROI, however, these small corpus callosum areas are simply a group of pixels with similar signal intensity, where the directional property of its originating tract is no longer visible (see Fig 2B). With the assistance of image analysis using our power spectrum method, we detected distinct alignment profiles of individual ROIs. Following polar conversion, these ROIs showed clear dominance of an orientation peak that dictated the dominant aligning angle of specific white matter tracts passing through correspondent ROIs. Moreover, while this study is targeting the NAWM, this method can be used to assess different tissue structures such as the gray matter, which is expected to show increased complexity of angular profile (data not shown) resulting from increased combination of nerve structures.

Using the corpus callosum, we show that the dominant orientation of each ROI calculated using FT power spectrum is consistent with the predicted directions of the corpus callosum at individual ROI regions. This is similar for both patients and controls. In this study, the angular values calculated directly from the ROI were frequency-domain orientations. After reciprocal conversion based on the FT theorem, the new orientation values were used to compare with projected directions. We found that the right genu and splenium show the best consistency, while the central region of genu and splenium showed the largest variability (Fig 4). The observation of similarity in dominant aligning angles between patients and controls is reasonable because both cohorts share a similar pattern of fiber projection in the corpus callosum. Moreover, the exclusion of lesions involving the corpus callosum may also play a role in the uniformity of this sample. Nonetheless, as MS patients are associated with greater NAWM damage than controls [18], the complexity of tissue alignment should also be higher in patients.

The degree of tissue damage in this study is evaluated using a new quantitative outcome, angular entropy. Measurement of entropy has been shown to be a powerful tool for assessing tissue injury, although most previous studies have been focusing on the traditional calculation of entropy that is based on the complexity rather than aligning directions of image features. Using a statistical assessing method, Theocharakis et al have shown that sum of entropy in signal intensity is one of the best variables to differentiate MS lesions from microangiopathy in the brain using T_2_-weighted MRI [19]. Based on wavelet transform, Zhang et al [20] demonstrate that the entropy of wavelet sub-bands can help enhance the detectability of MS tissue from healthy controls. Currently, with routine MRI, assessment of entropy based on the aligning property of a tissue structure is scarce, making angular entropy a valuable complementary index. In contrast to traditional calculations, angular entropy takes account of the number, distribution, and dominance of the aligning directions contained in a tissue. This makes it particularly useful to detect subtle changes in white matter because nerve tracts are typically highly aligned. Indeed, in a recent histological study, researchers demonstrate that demyelination is associated with significantly greater angular entropy than the intact myelin in mouse spinal cord [21]. In the present study, we showed that angular entropy is also significantly elevated in MS patients as compared with controls using T2-weighted MRI, that of considerably lower resolution than histological images. These findings suggest that higher angular entropy (less negative values in this study) is associated with greater tissue complexity, and therefore more severe nerve damage. This is further reflected in the SPMS patients who tended to show the highest angular entropy as compared to RRMS and control subjects in the corpus callosum.

Despite the encouraging findings, we note some limitations in this study. This was a proof-of-concept study, and therefore we were mainly focused on a highly organized structure, corpus callosum. As the largest inter-hemispheric white matter structure, corpus callosum is a frequent site of MS pathology [22]. Moreover, the anatomical trajectory of fiber tracks in the corpus callosum has already been validated previously in post-mortem studies [16], making this structure an ideal model for evaluating fiber track outcomes [23]. In this study, we positioned the ROIs in areas of corpus callosum with visually predictable orientation of fiber tracks. This may raise the suspicion of reliability of the ROIs. However, it is worth noting that although the alignment of white matter tracks in the corpus callosum when visualized as a whole is predictable in brain MRI, the pattern of alignment of individual MRI pixels is not identifiable visually in any arbitrary ROIs, either in the image (Fig 1B) or in the pre-processed FT domains (Fig 1C). In fact, in the process of ROI determination, the only confounder was the ROI size, for which we made sure that each ROI was located within the boundary of corpus callosum to avoid partial volume effect. In addition, the sample size of this study was small, which may contribute to the variability between ROIs and limit the degree of significance of the results. Furthermore, while the outcome measures are sensitive to tissue alignment, the contribution of specific pathologies to these measurements is subject to further confirmation. In the future, we seek to investigate how tissue orientation measures help understand disease progression, and how our method compares with other image assessing methods such as machine learning [24] and with advanced MRI techniques including diffusion-tensor imaging [5] and structure tensor analysis [25].

## 5. Conclusions

We have shown that it is possible to characterize the alignment of white matter tracks using clinical MRI through quantitative analysis of the FT power spectrum. This would be critical for improving the evaluation of disease activity in normal appearing tissues such as the corpus callosum because NAWM pathology is shown to play a significant role in the progression of disability in MS patients. With further confirmation, this method may also be used to evaluate tissue repair following injury in patients with or without treatment. Moreover, the ROIs we assessed in this study have a similar size to that of typical MS lesions in MRI. Therefore, assessing lesion injury and repair may be also possible using this method. In the clinical management of MS patients, we can compute the dominant orientation and angular entropy of select areas such as corpus callosum, or of focal lesions that are new or that show changes over time to detect invisible characteristics of tissue coherency. This information can be obtained at a secondary workstation and serve as valuable add-ons to the routine clinical reports that focuses mostly on lesion size, number, or volume. Finally, while this study is focused on MS, given its image post-processing nature, this method is applicable to other white matter diseases that involve disruption of tissue alignment and coherency.
